# Time Points for Gonadotropin-Releasing Hormone Stimulation Test Results in Korean Children

**DOI:** 10.3390/jcm10020252

**Published:** 2021-01-12

**Authors:** Rihwa Choi, Aerin Kwon, Youngju Oh, Sang Gon Lee, Eun Hee Lee

**Affiliations:** 1 Department of Laboratory Medicine and Genetics, Samsung Medical Center, Sungkyunkwan University School of Medicine, Seoul 06351, Korea; rihwa.choi@gclabs.co.kr; 2 Department of Laboratory Medicine, Green Cross Laboratories, Yongin, Gyeonggi 16924, Korea; aerinkwon@gclabs.co.kr (A.K.); yjoh@gclabs.co.kr (Y.O.); 3 Green Cross Laboratories, Yongin, Gyeonggi 16924, Korea

**Keywords:** precocious puberty, GnRH stimulation, luteinizing hormone, Korea, children

## Abstract

The gold standard for the laboratory diagnosis of central precocious puberty is based on the measurement of luteinizing hormone (LH) after gonadotropin-releasing hormone (GnRH) stimulation. We sought to investigate the laboratory data for GnRH stimulation testing using samples collected from Korean children at different time points. Sampling times were at the basal time point (0) and 15, 30, 45, 60, 90, and 120 min after GnRH stimulation. Pubertal response was defined as occurring when the peak LH concentration was 5 IU/L or more and rose to at least 2 times the basal LH concentration after GnRH stimulation. During the study period, 19,990 test results from 1958 Korean children (1841 females aged 1.3–8.9 years and 117 males aged 7.3–9.9 years) were obtained. Among the 1958 children, 1232 (62.9%) showed pubertal responses. The receiver operating characteristic curve that demonstrated the greatest area under the curve (AUC) among all examined time points was 45 min after GnRH stimulation in males (AUC 0.982, 95% CI 0.938–0.998) and 60 min in females (AUC 0.975, 95% CI 0.967–0.981). The combination of 45 min and 60 min showed the greatest AUC (0.996, 95% confidence interval 0.991–0.998), with a sensitivity level of 99.1% and a specificity of 100% for all children. The results of this study provide a possibility for a reduction in sampling time points (45 min and 60 min) to identify the presence of a pubertal response after GnRH stimulation in Korean children.

## 1. Introduction

Gonadotropin-releasing hormone (GnRH) stimulation tests are used to identify central precocious puberty in children [1]. Puberty results when the pulsatile secretion of GnRH is initiated and the hypothalamic–pituitary–gonadal axis is activated [2]. Thus, the most common cause of progressive precocious puberty (either central (CPP) or gonadotropin-dependent precocious puberty) is the early activation of pulsatile GnRH secretion, which may result from the presence of hypothalamic tumors or lesions [1,2]. In most cases, however, the cause remains unexplained [1,2]. Clinical findings related to CPP are very similar to those for physiological pubertal development, other than chronological age—younger than the normal age range of onset of puberty is (i.e., before eight years in girls and nine years in boys) [1,3]. It is important to distinguish between CPP and common variants of precocious puberty, such as premature thelarche (defined by the isolated development of breast tissue, without any other pubertal findings, which usually regresses over several months) or premature adrenarche (characterized by pubic or axillary hair growth secondary to mildly increased concentrations of adrenal-derived androgens without accompanying breast or testicular enlargement) [1,2,3]. However, early breast or testicular development is not always a manifestation of CPP, and clinical and laboratory investigations can help to resolve the diagnosis [1]. In South Korea, parents and their children visit pediatric clinics and undergo evaluation for CPP because of concerns including early menarche in girls and short adult stature due to early epiphyseal fusion and adverse psychosocial outcomes in both sexes [4,5].

Although baseline random luteinizing hormone (LH), GnRH-stimulated follicle-stimulating hormone (FSH), the LH to FSH ratio, levels of testosterone, estradiol, and other hormones, and hormonal binding protein profile analysis can be used in the evaluation of precocious puberty, the gold-standard for biochemical diagnosis involves the assessment of LH after stimulation with exogenous LH-releasing hormone (LHRH), GnRH, or GnRH agonists [2,6]. Although different stimulation test protocols have been described, and it has been reported that LH levels vary according to the assay used, the most commonly used stimulated peak LH concentration cut-off value is 5 IU/L [1,2]. This test requires blood sampling to be performed at one or more time points for gonadotrophin measurement at a time point ranging from 15 to 180 min after intravenous or subcutaneous GnRH administration [1,3,7].

In South Korea, GnRH stimulation test results are required to diagnose and treat CPP with therapeutics in accordance with a national notification (notification number 2017-180) released by the Ministry of Health and Welfare of Korea regarding the use of GnRH agonists for treatment of CPP [8]. Multiple time-point blood sampling is comparatively expensive, time consuming, and uncomfortable for patients, and there have been many attempts to simplify this test, e.g., by determining the LH concentration in only a single blood sample obtained at a selected time point [7,9]. However, there are no guidelines available regarding the best time point for measurement [3,6,8,9,10]. The Korean Society of Pediatric Endocrinology’s Clinical Guidelines for precocious puberty recommend the consecutive measurement of LH at intervals of 15–30 min from basal conditions to 90 to 120 min after the application of a standard dose of LHRH (100 µg) via injection for GnRH stimulation [8]. The guidelines do not specify exact time points for LH measurement after GnRH stimulation but, rather, allow clinicians to select time points.

Furthermore, limited studies are available regarding the best time point for blood sampling after GnRH stimulation in Korean children [7,11,12,13,14]. Previous studies reported differences in hormone concentration ranges of LH and FSH in different ethnic populations using different analytical platforms [15,16]. Previous studies conducted to evaluate the best time point for blood sampling after GnRH agonist application in populations other than Korean used different doses of GnRH agonist, limited numbers of male subjects, different LH cut-off values, and different analytical platforms to diagnose CPP [6,17,18,19].

Previous studies regarding the prevalence of a pubertal response in Korean children who have undergone a GnRH stimulation test and/or time points for blood sampling after GnRH stimulation in Korea included female subjects only or used results from methods other than the electrochemiluminescence immunoassay (ECLIA) [7,11,12,13,14]. Due to the pulsatile nature of GnRH secretion and the corresponding fluctuating gonadotropin values, which are influenced by pre-analytical, analytical (type of assay), and biological factors that may be different in diverse ethnic populations and sexes, studies including a large number of Korean children, both boys and girls, and data from various analytical platforms are needed [15,16].

Therefore, in this study, we sought to evaluate GnRH stimulation test results through serial sampling at different time points to elucidate the prevalence of a pubertal response in Korean children who had undergone a GnRH stimulation test due to the suspicion of precocious puberty. We also aimed to determine which time points for blood sampling are ideal for the assessment of time to achieve the peak concentration of LH as well as concentrations over and below 5 IU/L after GnRH stimulation for the diagnosis of CPP in Korean children.

## 2. Materials and Methods

### 2.1. Study Populations

From September 2017 to December 2018, GnRH stimulation test results from Korean females younger than eight years and 365 days, and from males younger than nine years and 365 days who visited one of 18 private pediatric clinics for LH measurement after GnRH stimulation were obtained from the laboratory information system of Green Cross Laboratories. Green Cross Laboratories is one of the largest referral clinical laboratories in Korea. When duplicate tests existed, only the patient’s first results were included. Records missing age and sex data were excluded. All data were anonymized prior to analysis. Information about the GnRH stimulation test could not be evaluated because this retrospective study used anonymized laboratory test results. During the study period, 19,990 test results detailing the LH concentration after GnRH stimulation from 1958 Korean children (1841 females aged 1.3–8 years and 365 days, and 117 males aged 7.3–9 years and 365 days) were obtained. This study was conducted in accordance with the guidelines laid down in the Declaration of Helsinki, and all procedures involving human subjects were approved by the institutional review board of Green Cross Laboratories (GCL-2019-1010-04). The datasets generated and analyzed during the current study are available from the corresponding authors upon reasonable request.

### 2.2. Definitions

Pubertal response was defined as a peak LH hormone level of 5 IU/L or more, and more than 2 times the basal LH concentration after GnRH stimulation, according to previous literature and a national notification (2017-180) released by the Ministry of Health and Welfare of Korea regarding the use of GnRH agonists for the treatment of CPP [4,7,8,11,12,13,14]. Girls aged less than 8 years and 365 days, and boys aged less than 9 years and 365 days were included [4,7,8,11,12,13,14]. It has been reported that CPP patients show a pubertal response during GnRH stimulation testing [4,7,8,11,12,13,14]. The other participants were defined as showing a prepubertal response.

### 2.3. Analytical Procedures

Serum LH and FSH concentrations were measured using an ECLIA assay with Elecsys LH and Elecsys FSH (Roche Holding AG, Basel, Switzerland) in accordance with the manufacturer’s instructions. The limit of quantification for both the LH and FSH assays was 0.3 IU/L. The percentage coefficient of variation for the replicate analysis was 3.6% for the LH assay and 3.7% for the FSH assay in the range of 0.3 to 200 IU/L.

### 2.4. Statistical Analysis

Categorical variables are presented as frequencies and percentages. The chi-square test was used to compare categorical variables. LH and FSH levels below the lower limit of quantification (0.3 IU/L for LH and 0.3 IU/L for FSH) were replaced by the value of the lower limit of quantification for the statistical analysis. We used nonparametric methods when data (continuous variables) were not normally distributed. To investigate the diagnostic value of LH at different time points after GnRH stimulation, we performed a receiver operating characteristic (ROC) curve analysis. Statistical analysis was executed using MedCalc software for Windows, version 19.1.3 (MedCalc Software bv, Ostend, Belgium). *p* values were considered to be significant at a level of 0.05.

## 3. Results

The characteristics of the study population of 1958 Korean children are summarized in Table 1. Among 1958 children, 1232 children (62.9%) had pubertal responses (peak LH concentration ≥ 5 IU/L and more than 2 times the basal LH). Age, basal LH and FSH concentrations, peak LH and FSH concentrations, and the peak LH/FSH ratio were significantly different between the pubertal- and prepubertal-response groups (*p* < 0.05). The prevalence of children with a pubertal response was not significantly different between males and females (*p* = 0.064).

**Figure 1:**
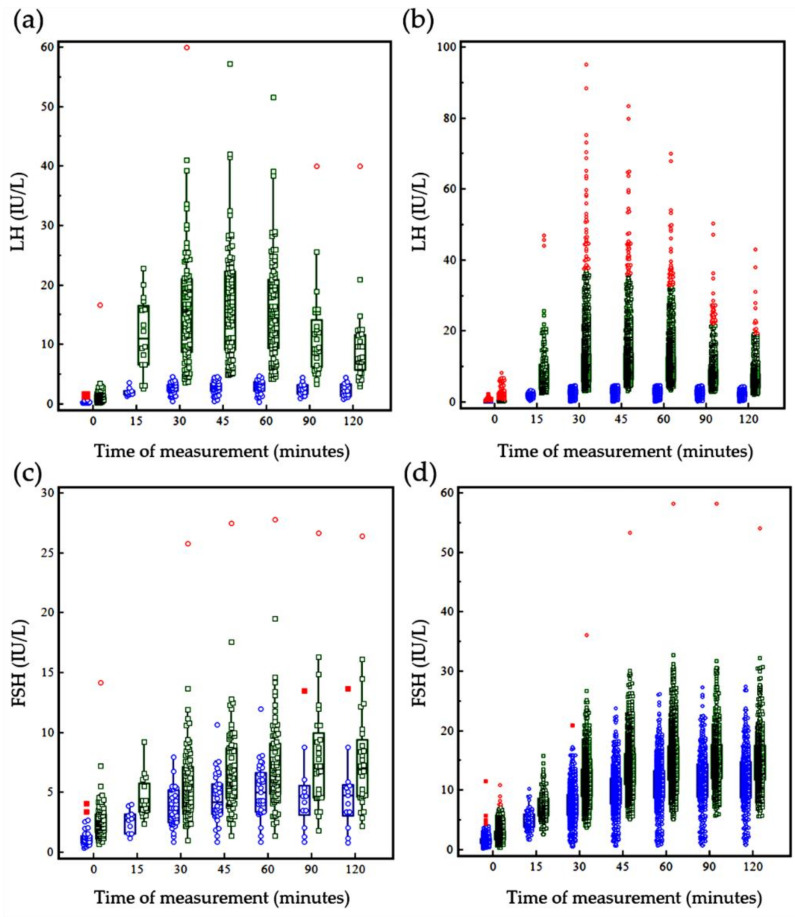
Gonadotropin-releasing hormone (GnRH)-stimulated hormone results for the pubertal-response group and the prepubertal-response group using the cutoff value of 5.0 IU/L or more and more than 2 times the basal LH concentration. (**a**) LH concentration in males; (**b**) LH concentration in females; (**c**) FSH concentration in males; (**d**) FSH concentration in females. In the figure, the central boxes represent the values from the 25–75 percentiles, and the middle line represents the median. Blue circles represent data from the prepubertal response group, and green squares represent data from the pubertal response group. Lines extend from the minimum to the maximum values, excluding outliers smaller than the lower quartile minus 1.5 times the interquartile range or larger than the upper quartile plus 1.5 times the interquartile range, known as inner fences, as well as values smaller than the lower quartile minus 3 times the interquartile range or larger than the upper quartile plus 3 times, known as outer fences. Outliers are plotted in red.

Different sampling times were incorporated during hormone analysis after GnRH stimulation, including the basal time point (0) and 15, 30, 45, 60, 90, and 120 min after GnRH stimulation. Hormone levels at each time point after GnRH stimulation are shown in Figure 1 and Table 2. The cumulative frequency of the pubertal response (time taken to achieve an LH concentration ≥ 5 IU/L and more than 2 times the basal LH), the time taken to achieve the peak LH concentration, and the time taken to decrease the LH level to below 5 IU/mL at each sampling time point during GnRH stimulation testing are summarized in Table 3. In 83 males with pubertal responses, it took 15 to 30 min to achieve more than 2 times the basal LH concentration and ≥5 IU/L. It was observed that the time taken to reach the peak LH concentration was within 60 min in all males with a pubertal response but one (one boy had an LH concentration of 5 IU/L at 30 min and showed a peak LH concentration at 120 min after GnRH stimulation). In females, the time range was more varied than that seen in males. Among all children, 97.4% of males (114/117) and 96.6% of females (1779/1841) achieved their peak LH concentration within 60 min. Among the 1232 children who showed a pubertal response, 258 (20.6%) showed a decrease in the LH concentration of below 5 IU/L after reaching the peak concentration. Of note, it took 45 to 120 min to achieved the decreased LH level of below 5 IU/L in all but one of these 258 children.

**Figure 2:**
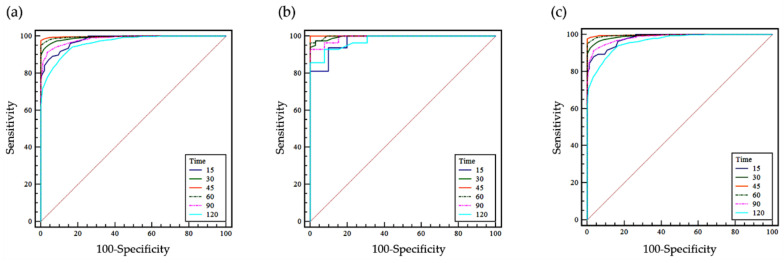
Receiver operating characteristic (ROC) curves of the LH at the basal time point (0) and 15, 30, 45, 60, 90, and 120 min after GnRH stimulation in (**a**) all patients, (**b**) males, and (**c**) females. The ROC curve analysis was performed to identify the best time point at which to differentiate pubertal responses using quantitative data for LH measurements.

The ROC curves of LH at the different time points (basal (0) and 15, 30, 45, 60, 90, and 120 min after GnRH stimulation) are summarized in Figure 2 for all patients. The ROC curve for LH at 45 min after GnRH stimulation had the greatest area under the curve (AUC) in males, and that at 60 min after GnRH stimulation had the greatest AUC in females (Table 4). An additional ROC curve analysis for the combination of two time points showed that the combination of 45 min and 60 min produced the greatest AUC for both males and females (Table 4).

## 4. Discussion

In this study, we evaluated the LH test results after GnRH stimulation according to different time points after stimulation using automated ECLIA methods in Korean children. Because it has been reported that LH levels vary according to the assay used, and because there are limited data on the LH concentration after GnRH stimulation available in children collected using ECLIA methods, these updated data from a relatively large sample size provide useful information about when to conduct GnRH stimulation testing in Korean children with this assay [2]. Furthermore, previous studies performed in Korea used data from only girls (Table 5). In Korea, the incidence and prevalence (per 100,000 persons) of CPP in boys is less than that in girls (incidence is 262.8 in girls and 7.0 in boys; prevalence is 410.6 in girls and 10.9 in boys) [20]. The lower prevalence and incidence in boys than in girls have also been reported in other ethnic populations, such as in Danish and Spanish children [20]. However, there are limited data from boys regarding the ideal blood-sampling time for the diagnosis of CPP via the GnRH stimulation test [6,16]. The strength of this study is the inclusion of a relatively large dataset from Korean boys.

Previous studies performed in Korean girls using analytical methods other than ECLIA reported that 30 or 45 min was the best time point for blood sampling after GnRH stimulation [7,11,12,13,14]. In this study, the ROC curve analysis for this single time point showed that the greatest AUC value for the LH concentration in males occurred at 45 min, and this result had high levels of sensitivity and specificity. In females, LH measurement at 60 min achieved the greatest AUC value. More than 45 min was needed to achieve an LH concentration of 5 IU/L or more, and more than 2 times the basal LH concentration in four of the 1232 children who showed a pubertal response. In three of these four children, 120 min passed before the achievement of this LH concentration. Meanwhile, it took only 30 min to achieve an LH concentration of 5 IU/L or more, and more than 2 times the basal LH after GnRH stimulation in all males who showed a pubertal response. However, in females, the response rate was slow and more variable than that in males. In this study, data from more girls with a greater age range than boys were included, and the dispersion of the corresponding optimal times was greater in females than in males. It has been reported that fertility hormone change is complex and depends on age and sex during growth and puberty [15]. LH and FSH markedly increase during puberty in females, but this occurs to a lesser extent in males [15,16]. The results of this study are comparable with previous findings regarding the complexity and extent of hormone changes by age and sex, although clinical information is limited. Future studies including large numbers of subjects with different ages and corresponding clinical information are needed to clarify the different hormone changes that occur and to determine the optimal time points at which to measure LH after GnRH stimulation.

Results from a considerable (20.6%) portion of children with pubertal responses showed decreased LH concentrations below 5 IU/L at 45 to 120 min after GnRH stimulation, while LH concentrations were decreased below 5 IU/L at 45 min after GnRH stimulation in seven children in this study. Among 1177 patients with pubertal responses whose blood was drawn at 45 min, 26 (2.2%) would not have been identified as having a pubertal response in this study if a single 45-min sampling method was applied.

Although some previous studies discussed the use of two time points for blood sampling based on their results, they did not perform a statistical analysis using the ROC curve analysis with the combination of two time points [7,11,12,13,14,15,16]. An additional ROC curve analysis for the combination of two time points showed that the combination of 45 min and 60 min produced the greatest AUC in this study. These findings suggest that multiple sampling time points are needed to diagnose CPP. The difference in the best LH measurement time identified among studies might be due to differences in the analytical methods used to quantify LH, the injection conditions of GnRH for stimulation, and the cohort characteristics, such as sex and age [1,2]. In South Korea, a standard dose of GnRH (100 μg) is given to diagnose CPP, while other countries may use a GnRH dose that is weight-adapted or adapted to the body surface of patients [6]. Future studies are needed to identify the best timing for LH measurement after GnRH stimulation to diagnose CPP with consideration of these factors to reach a consensus [6,21].

The European Society for Paediatric Endocrinology and the Endocrine Society define Precocious puberty as puberty that starts before age 8 in girls and 9 in boys, definitions that are different from those used in the present study (8 years and 365 days for girls and 9 years and 365 days for boys, based on criteria for reimbursable CPP treatment in Korea) [8,10]. This difference may limit the generalizability of the results of this study to other ethnic populations.

The limitation of this study is the lack of details pertaining to the patients’ personal history and the conduct of physical examinations to assess puberty such as Tanner staging and other laboratory and imaging tests to diagnose CPP. The lack of information weakens the study and makes it less generalizable to children with clinical signs of CPP. However, because the gold standard for the laboratory diagnosis of CPP is based on LH measurements after GnRH stimulation [1], this study still has value. The results of this study may be generalizable to populations in which a standard dose of GnRH (100 μg) is used and LH is measured using the ECLIA method to diagnose CPP. Furthermore, the use of recently collected data from a relatively large number of Korean children who underwent GnRH-stimulated hormone testing gives strength to this study. However, future studies are needed to investigate appropriate GnRH stimulation test strategies to diagnosis CPP using a variety of clinical information and to elucidate the best time points for sampling in various ethnic populations using different measurement methods.

## 5. Conclusions

In conclusion, we investigated hormone results after GnRH stimulation testing to identify the presence of CPP in Korean children. Considering the time points necessary to achieve the peak LH concentration and to decrease the LH level below the cut-off value of 5.0 IU/L, multiple time points, including 45 min and 60 min, need to be assessed in relation to GnRH stimulation test outcomes for the ECLIA assay used in this study. The results of this study provide useful information about the clinical application of GnRH stimulation testing.

## Figures and Tables

**Table 1:** Basal characteristics and gonadotropin-releasing hormone-stimulated hormone results.

	Male (*n* = 117)				*p* *	Female (*n* = 1841)				*p* *
Variables ^a^	Prepubertal Response (*n* = 34)		Pubertal Response (*n* = 83)			Prepubertal Response (*n* = 692)		Pubertal Response (*n* = 1149)		
	Med	IQR	Med	IQR		Med	IQR	Med	IQR	
Age (years)	9.1	8.6–9.6	9.7	9.4–9.9	<0.001	8.2	6.2–8.8	8.4	7.9–8.8	<0.001
Basal LH	0.3	0.3–0.4	0.9	0.4–1.7	<0.001	0.3	0.3–0.3	0.3	0.3–0.4	<0.001
Basal FSH	1.2	0.8–1.4	2.3	1.7–3.2	<0.001	1.5	1.1–1.9	2.3	1.8–3.2	<0.001
Peak LH	3.3	2.4–3.9	16.7	9.7–22.8	<0.001	3.4	2.6–4.2	8.7	6.5–14.1	<0.001
Peak FSH	5.3	3.5–6.5	6.7	5.0–9.2	<0.001	11.5	9.0–14.3	14.9	12.3–18.1	<0.001
Peak LH/FSH ratio	0.6	0.5–0.8	2.2	1.3–4.1	<0.001	0.3	0.2–0.4	0.6	0.4–1.0	<0.001

Abbreviations: FSH, follicle-stimulating hormone; LH, luteinizing hormone; Med, median; IQR, interquartile range ^a^ Units for LH and FSH: IU/L. * *p*-values from the Mann-Whitney U test.

**Table 2:** Numbers and frequency (%) of test results at each sampling time point during the GnRH stimulation test.

	Basal	15 min		30 min		45 min		60 min		90 min		120 min	
	*n*	*n*	%	*n*	%	*n*	%	*n*	%	*n*	%	*n*	%
Male	117	26	22.2	117	100.0	116	99.1	115	98.3	41	35.0	41	35.0
Pubertal response group	83	16	19.3	83	100.0	82	98.8	82	98.8	28	33.7	28	33.7
First time to achieve >2 times the basal LH concentration and ≥5 IU/L		13	15.7	64	77.1	5	6.0	1	1.2	0	0.0	0	0.0
First time to reach peak the LH concentration		0	0.0	18	21.7	53	63.9	11	13.3	0	0.0	1	1.2
First time to decrease the LH concentration < 5 IU/L		0	0.0	0	0.0	1	14.3	3	42.9	0	0.0	3	42.9
Prepubertal response group	34	10	29.4	34	100.0	34	100.0	33	97.1	13	38.2	13	38.2
First time to reach the peak LH concentration	1	0	0.0	7	20.6	17	50.0	7	20.6	1	2.9	1	2.9
Female	1841	226	12.3	1839	99.9	1769	96.1	1834	99.6	979	53.2	934	50.7
Pubertal response group	1149	142	12.4	1147	99.8	1095	95.3	1148	99.9	582	50.7	555	48.3
First time to achieve >2 times the basal LH and ≥5 IU/L		90	7.8	926	80.6	113	9.8	17	1.5	1	0.1	2	0.2
First time to reach the peak LH concentration		1	0.1	414	36.0	598	52.0	118	10.3	7	0.6	11	1.0
First time to decrease the LH concentration < 5 IU/L		0	0.0	1	0.4	6	2.2	47	17.5	108	40.3	106	39.6
Prepubertal response group	692	84	12.1	692	100.0	674	97.4	686	99.1	397	57.4	379	54.8
First time to reach the peak LH concentration		0	0.0	75	10.8	391	56.5	182	26.3	20	2.9	24	3.5

**Table 3:** Number and cumulative frequency (%) of test results at each sampling time point during the GnRH stimulation test.

	Basal	15 min		30 min		45 min		60 min		90 min		120 min	
	*n*	*n*	%	*n*	%	*n*	%	*n*	%	*n*	%	*n*	%
Male	117												
Pubertal response group	83												
LH concentration reached >2 times the basal LH and ≥5 IU/L		16	19.3	77	92.8	82	98.8	83	100	0	100	0	100
Peak LH concentration reached		0	0	18	21.7	71	85.5	82	98.8	82	98.8	83	100
LH concentration decreased to <5 IU/L		0	0	0	0	1	14.3	4	57.1	4	57.1	7	100
Prepubertal response group	34												
Peak LH concentration reached	1	0	0	8	23.5	25	73.5	32	94.1	33	97.1	34	100
Female	1841												
Pubertal response group	1149												
LH concentration reached >2 times the basal LH and ≥5 IU/L		90	7.8	1016	88.4	1129	98.3	1146	99.7	1147	99.8	1149	100
Peak LH concentration reached		1	0.1	415	36.1	1013	88.2	1131	98.4	1138	99.0	1149	100
LH concentration decreased to <5 IU/L		0	0	1	0.4	7	2.6	54	20.1	162	60.4	268	100
Prepubertal response group	692												
Peak LH concentration reached		0	0	75	10.8	466	67.3	648	93.6	668	96.5	692	100

**Table 4:** Results of the ROC curve analysis performed to investigate the best time at which to conduct LH measurements after GnRH stimulation to identify the presence of a pubertal response.

	Total (*n* = 1958)				Male (*n* = 117)				Female (*n* = 1841)			
Rank	Time of Measure	AUC	SE	95% CI	Time of Measure	AUC	SE	95% CI	Time of Measure	AUC	SE	95% CI
1	45 & 60 min	0.996	0.001	0.991 to 0.998	45 & 60 min	0.994	0.006	0.958 to 1.000	45 & 60 min	0.996	0.001	0.991 to 0.998
2	30 & 45 min	0.991	0.002	0.986 to 0.995	30 & 45 min	0.994	0.006	0.958 to 1.000	30 & 45 min	0.991	0.002	0.986 to 0.995
3	30 & 60 min	0.987	0.002	0.980 to 0.991	30 & 60 min	0.988	0.008	0.948 to 0.999	30 & 60 min	0.987	0.002	0.980 to 0.991
4	45 & 90 min	0.986	0.002	0.980 to 0.991	45 & 90 min	0.988	0.008	0.948 to 0.999	45 & 90 min	0.986	0.002	0.980 to 0.991
5	45 & 120 min	0.985	0.002	0.979 to 0.990	45 & 120 min	0.988	0.008	0.948 to 0.999	45 & 120 min	0.985	0.003	0.978 to 0.990
6	60 & 120 min	0.976	0.003	0.968 to 0.982	45 min	0.982	0.010	0.938 to 0.998	60 & 90 min	0.976	0.003	0.968 to 0.982
7	15 & 60 min	0.975	0.003	0.967 to 0.981	15 & 45 min	0.982	0.010	0.938 to 0.998	60 & 120 min	0.976	0.003	0.968 to 0.983
8	60 & 90 min	0.975	0.003	0.967 to 0.982	30 & 90 min	0.982	0.010	0.938 to 0.998	60 min	0.975	0.003	0.967 to 0.981
9	60 min	0.974	0.003	0.966 to 0.981	15 & 60 min	0.976	0.012	0.929 to 0.995	15 & 60 min	0.975	0.003	0.967 to 0.981
10	15 & 45 min	0.968	0.004	0.959 to 0.975	60 min	0.970	0.013	0.920 to 0.993	45 min	0.966	0.004	0.957 to 0.974
11	45 min	0.967	0.004	0.958 to 0.975	30 & 120 min	0.970	0.013	0.920 to 0.993	15 & 45 min	0.966	0.004	0.957 to 0.974
12	30 & 90 min	0.956	0.004	0.946 to 0.964	60 & 90 min	0.970	0.013	0.920 to 0.993	30 & 90 min	0.954	0.004	0.943 to 0.963
13	30 & 120 min	0.951	0.004	0.941 to 0.960	60 & 120 min	0.970	0.013	0.920 to 0.993	30 & 120 min	0.950	0.004	0.939 to 0.959
14	15 & 30 min	0.944	0.005	0.932 to 0.953	30 min	0.964	0.014	0.912 to 0.990	30 min	0.942	0.005	0.930 to 0.952
15	30 min	0.943	0.005	0.932 to 0.953	15 & 30 min	0.964	0.014	0.912 to 0.990	15 & 30 min	0.942	0.005	0.930 to 0.952
16	15 & 90 min	0.730	0.007	0.710 to 0.750	15 & 90 min	0.735	0.028	0.645 to 0.812	15 & 90 min	0.730	0.007	0.709 to 0.750
17	90 & 120 min	0.690	0.007	0.669 to 0.710	15 & 120 min	0.717	0.027	0.626 to 0.796	90 & 120 min	0.692	0.007	0.671 to 0.713
18	90 min	0.689	0.007	0.668 to 0.710	15 min	0.705	0.027	0.613 to 0.786	90 min	0.691	0.007	0.670 to 0.713
19	15 & 120 min	0.679	0.007	0.657 to 0.699	90 min	0.657	0.026	0.563 to 0.742	15 & 120 min	0.676	0.007	0.654 to 0.697
20	15 min	0.655	0.007	0.633 to 0.676	90 & 120 min	0.657	0.026	0.563 to 0.742	15 min	0.651	0.007	0.629 to 0.673
21	120 min	0.637	0.006	0.615 to 0.659	120 min	0.639	0.025	0.545 to 0.725	120 min	0.637	0.007	0.615 to 0.659

Abbreviations: AUC, area under curve; CI, confidence interval; min, minutes; SE, standard error. This table ranks all individual time points and all possible combinations of two time points. AUCs were calculated to identify the best time point at which to differentiate pubertal responses using binary results based on a defined LH concentration (LH ≥ 5 IU/L and more than 2 times the basal LH).

**Table 5:** Studies performed to investigate the best time to measure LH after GnRH stimulation to diagnose central precocious puberty in children.

Reference	Study Region	Subjects	Analytical Methods	Investigated Measurement Times	Best Time to Measure LH	Criteria for Selection of the Best Time Point in the Study
Kandemir et al., 2011 [17]	Turkey	263 girls	CLIA, Architect system	Basal and after 20, 40, 60, and 90 min	40 min	Highest AUC (sensitivity 98%, specificity 100%)
Yazdani et al., 2012 [18]	USA	107 children 16 boys, 91 girls	CLIA, ADVIA Centaur System	Basal and after 1, 3, and 6 h	3 h	Highest cumulative frequency in CPP patients (100%)
Cavallo et al., 1995 [19] ^a^	USA	44 girls 7 boys	IMRA	Basal and after 15, 30, 45, and 60 min (Cincinnati) Basal and after 10, 20, 30, 45, and 60 min (Chicago)	Between 30–60 min ^a^	Visual inspection of ROC curves ^a^
Choi et al., 2007 [11]	Korea	45 girls	IMRA	Basal and after 15, 30, 60,90, and 120 min	30 min	Highest sensitivity to the diagnosis of CPP (sensitivity 100%, specificity 78.9%)
Kim et al., 2016 [12]	Korea	118 girls	IMRA	Basal and after 30, 45, and 60 min	30 min	Highest frequency in CPP patients (82.5%)
Yun et al., 2017 [13]	Korea	69 girls	IMRA	Basal and after 30, 60, 90, and 120 min	30 min	Highest cumulative frequency in CPP patients (100%)
Kim et al., 2015 [14]	Korea	72 girls	CLIA, Architect system	Basal and after 15, 30, 45, 60, 90, and 120 min	45 min	Highest cross-sectional (98.6%) and cumulative frequencies (100%)
Kim et al. 2011 [7]	Korea	166 girls	CLIA, ADVIA Centaur System	Basal and after 15, 30, 45, 60, 90, and 120 min	45 min	Highest cumulative frequency in CPP patients (100%)
This study	Korea	1958 children 117 boys 1841 girls	ECLIA, Cobas system	Basal and after 15, 30, 45, 60, 90, and 120 min	60 min (99.8%) ^b^ 45 min (100%) ^b^ 60 min (99.7%) ^b^	Highest AUC (sensitivity 95.0%, specificity 100%) Highest AUC (sensitivity 97.6%, specificity 100%) Highest AUC (sensitivity 97.8%, specificity 100%)

Abbreviations: AUC, area under the curve; CLIA, chemiluminescence immunoassay; CPP, central precocious puberty; ECLIA, electrochemiluminescent immunoassay; IMRA, immunoradiometric assay; min, minutes. ^a^ This study used a single LH cut-off value of 15 IU/L to distinguish CPP from non-CPP patients, and the best time to measure LH was taken from Cavallo et al.’s conclusion. ^b^ Cumulative frequency of pubertal responses based on the time of measuring the LH level among patients with a pubertal response (presented in parentheses).

## Data Availability

The datasets generated and analyzed during the current study are available from the corresponding authors upon reasonable request.

## References

[1] Latronico A.C., Brito V.N., Carel J.C. (2016). Causes, diagnosis, and treatment of central precocious puberty. Lancet Diabetes Endocrinol..

[2] Carel J.C., Leger J. (2008). Clinical practice. Precocious puberty. N. Engl. J. Med..

[3] Wei C., Davis N., Honour J., Crowne E. (2017). The investigation of children and adolescents with abnormalities of pubertal timing. Ann. Clin. Biochem..

[4] Rhie Y.J., Lee K.H. (2015). Overview and treatment of precocious puberty. J. Korean Med. Assoc..

[5] Kim S.H., Huh K., Won S., Lee K.W., Park M.J. (2015). A Significant Increase in the Incidence of Central Precocious Puberty among Korean Girls from 2004 to 2010. PLoS ONE.

[6] Krishna K.B., Fuqua J.S., Rogol A.D., Klein K.O., Popovic J., Houk C.P., Charmandari E., Lee P.A., Freire A.V., Ropelato M.G. (2019). Use of Gonadotropin-Releasing Hormone Analogs in Children: Update by an International Consortium. Horm. Res. Paediatr..

[7] Kim H.K., Kee S.J., Seo J.Y., Yang E.M., Chae H.J., Kim C.J. (2011). Gonadotropin-releasing hormone stimulation test for precocious puberty. Korean J. Lab. Med..

[8] The Korean Society of Pediatric Endocrinology (2011). The Korean Society of Pediatric Endocrinology’s Clinical Guideline for the Management of Precocious Puberty.

[9] Neely E.K., Hintz R.L., Wilson D.M., Lee P.A., Gautier T., Argente J., Stene M. (1995). Normal ranges for immunochemiluminometric gonadotropin assays. J. Pediatr..

[10] Carel J.C., Eugster E.A., Rogol A., Ghizzoni L., Palmert M.R., Antoniazzi F., Berenbaum S., Bourguignon J., Chrousos G.P., ESPE-LWPES GnRH Analogs Consensus Conference Group (2009). Consensus statement on the use of gonadotropin-releasing hormone analogs in children. Pediatrics.

[11] Choi J.-H., Shin Y.-L., Yoo H.-W. (2007). Predictive factors for organic central precocious puberty and utility of simplified gonadotropin-releasing hormone tests. Pediatr. Int..

[12] Kim J.-I., Kwon W.-H., Moon K.-C., Lee I.-W. (2016). Clinical Characteristics of precocious puberty girls and Comparison Analysis of GnRH Test results with Diagnosis type. J. Nucl. Med. Technol..

[13] Yun B.S., Kim K.H. (2017). Gonadotropin-Releasing Hormone Stimulation Test in Patients with Precocious Puberty. Korean J. Natl. Health Insur. Serv. Ilsan Hosp..

[14] Kim M.S., Hwang P.H., Lee D.-Y. (2015). A Gonadotropin-Releasing Hormone (GnRH) Stimulation Test Before and After GnRH Analogue Treatment for Central Precocious Puberty: Has the GnRH Test been Adequately Simplified?. Indian J. Pediatr..

[15] Adeli K., Higgins V., Trajcevsk K., Habeeb N.-W. (2017). The Canadian laboratory initiative on pediatric reference intervals: A CALIPER white paper. Crit. Rev. Clin. Lab. Sci..

[16] Higgins V., Fung A.W.S., Chan M.K., Macri J., Adeli K. (2018). Pediatric reference intervals for 29 Ortho VITROS 5600 immunoassays using the CALIPER cohort of healthy children and adolescents. Clin. Chem. Lab. Med..

[17] Kandemir N., Demirbilek H., Ozon Z.A., Gonc N., Alikasifoglu A. (2011). GnRH stimulation test in precocious puberty: Single sample is adequate for diagnosis and dose adjustment. J. Clin. Res. Pediatr. Endocrinol..

[18] Yazdani P., Lin Y., Raman V., Haymond M. (2012). A single sample GnRHa stimulation test in the diagnosis of precocious puberty. Int. J. Pediatr. Endocrinol..

[19] Cavallo A., Richards G.E., Busey S., Michaels S.E. (1995). A simplified gonadotrophin-releasing hormone test for precocious puberty. Clin. Endocrinol. (Oxf.).

[20] Kim Y.J., Kwon A., Jung M.K., Kim K.E., Suh J., Chae H.W., Kim D.H., Ha S., Seo G.H., Kim H. (2019). Incidence and Prevalence of Central Precocious Puberty in Korea: An Epidemiologic Study Based on a National Database. J. Pediatr..

[21] Brito V.N., Spinola-Castro A.M., Kochi C., Kopacek C., da Silva P.C.A., Guerra-Junior G. (2016). Central precocious puberty: Revisiting the diagnosis and therapeutic management. Arch. Endocrinol. Metab..

